# Outer membrane vesicles from *Escherichia coli* as a presentation platform for AR-23 antiviral peptide

**DOI:** 10.3389/fmolb.2025.1607578

**Published:** 2025-10-03

**Authors:** Francesca Mensitieri, Federica Dell’Annunziata, Giulia Gaudino, Veronica Folliero, Gianluigi Franci, Fabrizio Dal Piaz, Viviana Izzo

**Affiliations:** ^1^ Department of Medicine, Surgery and Dentistry “Scuola Medica Salernitana”, University of Salerno, Baronissi, Italy; ^2^ Department of Medicine, Surgery and Dentistry “Scuola Medica Salernitana”, Graduate School in Clinical Pathology and Clinical Biochemistry, University of Salerno, Baronissi, Italy; ^3^ University Hospital San Giovanni di Dio e Ruggi d’Aragona, University of Salerno, Salerno, Italy

**Keywords:** outer membrane vesicles (OMVs), antimicrobial peptides (AMPs), cytolysin A (ClyA), AR-23, presentation platforms, chimeric proteins, antiviral activity

## Abstract

**Introduction:**

Anti-microbial peptides (AMPs) are a well-established alternative among antiviral and antibacterial agents, having considerable advantages over traditional antimicrobials in terms of biocompatibility and limited resistance development. However, a general poor bioavailability and short half-life limit their large-scale implementation. In this framework, different strategies are being explored, such as AMPs encapsulation or their functionalization on antigen-presenting platforms. In this work the evaluation of *Escherichia coli* (*E. coli*) derived Outer Membrane Vesicles (OMVs) as antiviral presenting platforms is described.

**Methods:**

OMVs were engineered through the recombinant overexpression of an outer membrane chimeric protein, ClyA-AR23, obtained by combining Cytolysin A (ClyA) with the AR-23 antiviral peptide, derived from frog skin and active against herpes simplex viruses. LC-MS/MS was used to screen the presence of the recombinant protein in cells and OMVs. Plaque reduction assay after pre-incubation treatment and qPCR on viral transcript were used to evaluate ClyA-AR23 OMVs antiviral activity of the engineered vesicles.

**Results:**

The expression of ClyA-AR23 protein was verified in recombinant *E. coli* cells and OMVs and the surface exposure of ClyA C-terminus was confirmed. Engineered ClyA-AR23 OMVs negligible cytotoxicity effect was assessed on VERO-76 cells. Both control and functionalized OMVs were used in pre-incubation treatment with HSV-1, HSV-2, SARS-COV2 and PV-1. Results highlighted that ClyA-AR23 OMVs did effectively impair HSV-1 and HSV-2 replication cycle in a dose dependent manner.

**Discussion:**

In this work we provided a first evidence of AMPs functionalization on membrane vesicles of bacterial origin. The systems demonstrated to be active towards HSV-1 and HSV-2 viruses with negligible cytotoxicity on VERO-76 cells.

## 1 Introduction

Antimicrobial peptides (AMPs) are among the most promising therapeutic agents identified, developed and optimized over the last decade as potential antimicrobial treatments (Pane et al., 2016; Pane et al., 2017). These peptides, typically composed of 10–100 amino acids, are produced by all living organisms, in diverse ecological niches and serve as a first line of defense against pathogens (Bulet et al., 1999; Tang et al., 2018). Many different AMPs have been identified and described in literature for their antibacterial, antiviral, antiparasitic and antifungal activities. AMPs offer several advantages over traditional antibiotics, including the ability to simultaneously target multiple biological pathways (Eckert, 2011); in addition, they are characterized by a significantly reduced toxicity and a limited tendency to induce resistance mechanisms (Magana et al., 2020). AMPs main action mechanism lies in their ability to permeabilize negatively charged bacterial membranes, leading to insertion of AMPs into membrane leaflets, pore formation and subsequent cell lysis (Luo and Song, 2021). This activity has been proven effective against bacteria, fungi and enveloped viruses. Furthermore, synergy between AMPs and antibiotics has been observed, with significantly reduced minimum inhibitory concentrations (MIC) for antimicrobial drugs when combined with AMPs (Mhlongo et al., 2023), which are also endowed with the ability to modulate host immune response and regulate inflammation (Luo and Song, 2021; Mookherjee and Hancock, 2007).

Recently, AMPs derived from amphibian skin secretions have raised interest due to their marked antiviral activity. Frogs release AMPs from their dermal glands via a holocrine mechanism (Zannella et al., 2022; Varga et al., 2019). These AMPs represent a heterogeneous class of peptides, characterized by specific features: *i* the presence of basic amino acids, imparting a net positive charge to the molecule at neutral pH; *ii* the presence of approximately 50% hydrophobic amino acids; and *iii* the ability to assume alpha-helix or beta-sheet conformation when interacting with the target cell membrane (Zannella et al., 2022; Chianese et al., 2023). The Ranidae family, including *Rana temporaria*, *Rana tagoi*, *Rana muscosa*, and *Rana sakuraii*, are the major sources of amphibian AMP. Notably, a peptide isolated from *R*. *tagoi*, AR-23, was identified in 2003 by Conlon and coworkers. This 23-amino acid amphipathic alpha-helix peptide shares significant sequence similarity with melittin peptide (MRP) but features three positively charged residues at the C-terminus, responsible for boosting its activity (Urban et al., 2007; Zhang et al., 2016), rendering this peptide effective against HSV-1, HSV-2, Influenza virus, flaviviruses, and HIV-1 (Memariani et al., 2020).

Chianese et al investigated the antiviral activity of AR-23 towards HSV-1 where the peptide showed its highest antiviral activity. Their findings revealed that AR-23 disrupts the initial stages of herpes infection by interfering with viral attachment and entry into the host cell (Chianese et al., 2022). Noteworthy, AR-23 exhibited enhanced anti-HSV-1 activity when pre-incubated with the virus, suggesting its direct interaction with viral particles to block early infection stages (Chianese et al., 2022). This effect was observed for all enveloped viruses (HSV-1, Measles virus, Human parainfluenza virus type-2, HCoV-229E, SARS-CoV-2) but not for non-enveloped viruses (Poliovirus type 1), suggesting that AR-23 may interact with the viral envelope.

Despite their clear advantages as antimicrobial agents, the therapeutic use of AMPs is limited by poor bioavailability, short half-life and rapid biodegradation *in vivo* due to clearance and protease degradation; moreover, factors like serum ionic strength may be responsible of drastically reducing AMPs stability (Jabeen et al., 2023). As a result, various strategies, including encapsulating AMPs in drug delivery systems, are currently being explored to enhance their effectiveness and broaden their applicability (Drayton et al., 2020). AMPs delivery has been investigated through different approaches, aimed at improving AMPs stability, half-life, biocompatibility and pharmacokinetics. Some examples include the use of polydimethylsiloxane (PDMS) surface modification with a coating of an AMP (RRP9W4N)-functionalized cross-linked hydrogel, the encapsulation of HHC-8 and MM-10 AMPs in poly (ε-caprolactone) nanoparticles (PCL-NPs, and the immobilization of melittin AMP peptide on chitosan thin layers to obtain an efficient bactericidal surface. These functionalized nanoparticles exerted a high antibacterial effect against *S. epidermidis*, *S. aureus* and MRSA strains (Stepulane et al., 2022; Stepulane et al., 2024; Sharma et al., 2021; Zarghami et al., 2021).

For AMPs with antiviral activity, however, only a few studies have explored targeted drug delivery systems or immobilization on antigen-presenting platforms, and these strategies are still under development (Jabeen et al., 2023). Gabriel and coworkers obtained the conjugation of the human AMP LL37 onto cellulose beads, demonstrating its ability to inactivate the enveloped murine cytomegalovirus (Gabriel and Holtappels, 2021). Similarly, p41, a charged analogue of antiviral peptide C5A, was coated on polyamino acidic polymers. In this condition, p41 was protected from proteolysis and its haemolytic activity was nearly eliminated. Furthermore, p41 nanoparticles enhanced the suppression of HIV and HCV infections (Zhang et al., 2013). These works suggest that the immobilization of antiviral peptides might be a promising strategy to pursue in the development of novel antiviral treatments (Makowski et al., 2019).

OMVs, naturally produced by Gram-negative bacteria, are drawing much attention as versatile drug delivery systems. OMVs, ranging from 20 to 250 nm, are formed by the bulging of bacterial outer membrane and carry several components from the membrane, cell wall, periplasm and cytoplasm (Schwechheimer and Kuehn, 2015; Dell'Annunziata et al., 2024). OMVs are physiologically produced by bacteria to carry out a wide range of functions, including communication, delivery of biomolecules, as well as pathogenesis, biofilm formation and nutrient acquisition (Kulp and Kuehn, 2010; Dell'Annunziata et al., 2021; De Lise et al., 2019). All these features, along with the ease of engineering and the natural ability of OMVs to deliver biomolecules across cell membranes, make them promising candidates as drug delivery vehicles and as platforms for surface display (Collins and Brown, 2021). Different membrane proteins are currently used as membrane-anchoring proteins to immobilize antigens on OMVs surface. Among others, Cytolysin A (ClyA), a transmembrane α-pore-forming toxin produced by some Enterobacteriaceae, is one of the most exploited, and different works demonstrate its versatility for surface display in OMVs (Kim et al., 2008).

In this study, we describe the surface functionalization of *E. coli* derived OMVs with the AR-23 antimicrobial peptide. A chimeric construct bearing AR-23 peptide fused at the C-terminus of ClyA was recombinantly expressed in *E. coli* cells and the presence of the ClyA-AR23 chimeric protein was verified in both recombinant cells and OMVs by LC-MS/MS. The surface exposure of ClyA C-terminus was verified using the scaffold ClyA protein fused with a C-terminal his tag and immunofluorescence and limited proteolysis experiments were carried out. Finally, native and recombinant OMVs were used in antiviral pre-treatment assays against HSV-1, HSV-2 and SARS-COV2 enveloped viruses. Poliovirus (PV-1) was used as a non-enveloped virus and the biological activity of OMVs decorated with AR23 was characterized, allowing us to confirm the construction of a novel antiviral display platform, efficient against HSV viruses.

## 2 Results

### 2.1 Recombinant expression of ClyA-AR23 in *Escherichia coli* BL21 (DE3) and proteomic characterization of cell fractions and OMVs

**FIGURE 1:**
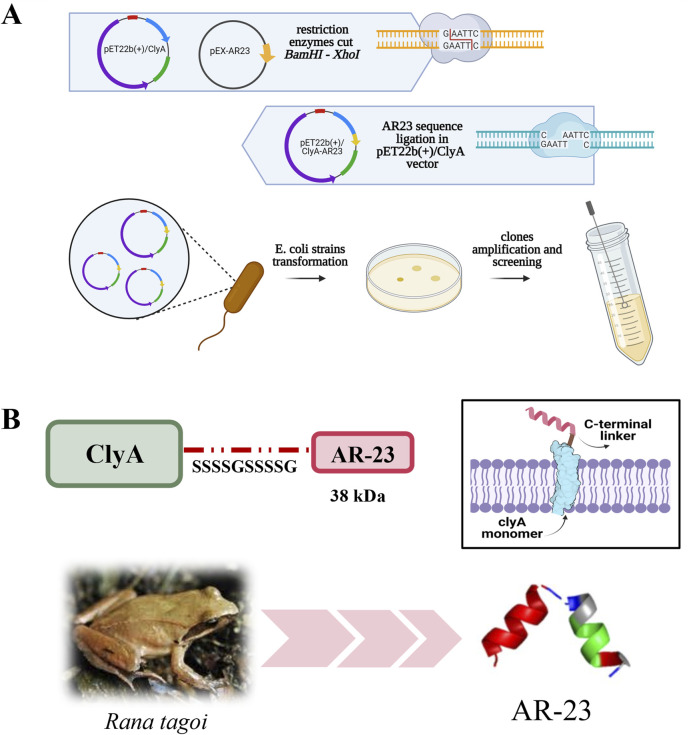
ClyA-AR23 cloning strategy and chimeric protein. **(A)** pET22b (+)/ClyA-AR23 cloning strategy. **(B)** ClyA-AR23 chimeric protein scheme and expected structure in the outer membrane, along with AR-23 hypothetical three-dimensional model (derived from PEP-FOLD3 software) are shown.

Plasmid pET22b (+)/ClyA-AR23 was obtained as described in the Materials and Methods section, with the cloning strategy outlined in Figure 1, which also includes a three-dimensional model of AR23, obtained with PEP-FOLD3 software (https://bioserv.rpbs.univ-paris-diderot.fr/services/PEP-FOLD3/), and a representation of the expected structure of ClyA-AR23 in the outer membrane. Recombinant ClyA-AR23 protein was expressed, OMVs were purified via ultrafiltration and ultracentrifugation. Cell paste of control (transformed with pET22b (+) plasmid) and recombinant *E. coli* cells after the recombinant expression were sonicated and the soluble and insoluble fractions of cell lysates were recovered after centrifugation. Protein concentration was determined by Bradford assay, followed by SDS-PAGE analysis.

**FIGURE 2:**
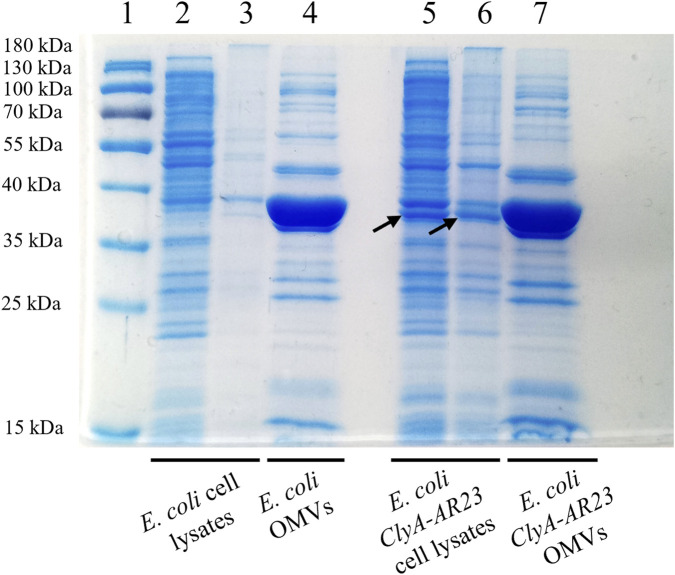
SDS-PAGE analysis of *Escherichia coli* BL21 (DE3) cell lysates and OMVs, control and recombinant strains. Lane 1 = MW standard, lane 2 = soluble fraction of *Escherichia coli* control strain cell lysate, lane 3 = insoluble fraction of *Escherichia coli* control strain cell lysate, lane 4 = OMVs of *Escherichia coli* control strain, lane 5 = soluble fraction of ClyA-AR23 expressing *Escherichia coli* strain cell lysate, lane 6 = insoluble fraction of ClyA-AR23 expressing *Escherichia coli* strain cell lysate, lane 7 = OMVs of ClyA-AR23 expressing *Escherichia coli* strain.

The SDS-PAGE analysis of the soluble and insoluble fractions of induced cultures, along with OMVs samples, is shown in Figure 2. A protein band corresponding to the expected molecular weight of ClyA-AR23 (37 kDa) is evident in both soluble and insoluble fractions of recombinant cells (black arrows in Figure 2) but absent in control samples. No significant differential protein bands were detected in recombinant OMVs compared to control OMVs (lanes 4 and 7 in Figure 2). However, a predominant band present in OMV fractions between 40 and 35 kDa, due to the presence of outer membrane proteins confirmed by proteomic analysis (data not shown), potentially hid underlying differences. Gel bands at different molecular weights were excised and processed for proteomic analysis.

**FIGURE 3:**
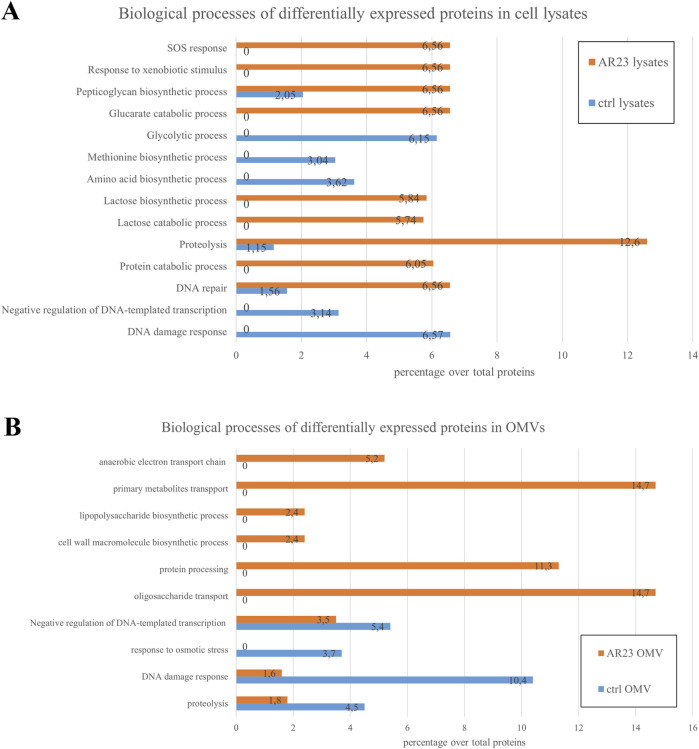
Biological processes of differentially expressed proteins in cell lysates and OMVs. **(A)** Cell lysates differential protein functions. **(B)** OMVs differential protein functions. Percentage over total proteins is shown.

The analysis of proteomic data was carried out using Mascot and Proteome Discoverer software. Proteins identified with significant scores were subjected to Gene Ontology analysis. Approximately 1,100 proteins were found for cell lysates and around 230 for OMVs, both in control and AR23-expressing cells. Comprehensive functional protein association networks analysis, performed by STRING software, is shown in Supplementary Figure S1. Overlapping protein functions in control and recombinant strains, involving metabolic pathways like sugar, amino acid, and cell wall biogenesis in cells were found (Supplementary Figures S1A,C). In addition, common functional features between control and recombinant OMVs included cell outer membrane homeostasis, peptidoglycan metabolism and transport systems (Supplementary Figures S1B,D). In ClyA-AR23 OMVs, protein export and galactose catabolism pathways were more represented compared to control OMVs. Although most protein functions overlapped between control and recombinant cells, we focused on differentially expressed proteins to identify proteomic changes potentially driven by recombinant expression. Proteins enriched in either control or recombinant samples were selected based on the significance of their abundance ratio retrieved in PD analysis (p-value <0.05), for both cell lysates and OMVs. Gene ontology analysis of these differentially expressed proteins was performed using FunRich software (FunRich 3.1.3) and is presented in Figure 3. Data shown in Figure 3A highlighted the primary biological processes differing between control and recombinant cell lysates. In negative control cells, the most abundant functions were involved in glycolysis and amino acid biosynthesis. Negative regulation of transcription and DNA damage response were also found. Conversely, ClyA-AR23 expressing cells displayed a higher abundance of proteins involved in lactose metabolism, protein catabolism and stress response.

These biological functions are likely related to the recombinant expression itself. Indeed, the addition of IPTG as an inducer and overstimulation of the protein production machinery are associated with these processes. DNA repair and peptidoglycan metabolism were also found to be enriched in recombinant cells patterns. Figure 3B reports biological functions differentially enriched in OMVs. Recombinant OMVs showed an enrichment in proteins involved in carbohydrate metabolism and transport, cell wall organization and protein processing. Instead, control OMVs displayed a higher abundance of proteins mainly implicated in osmotic stress response and DNA damage.

### 2.2 Nano-tracking analysis of control and recombinant OMVs

**FIGURE 4:**
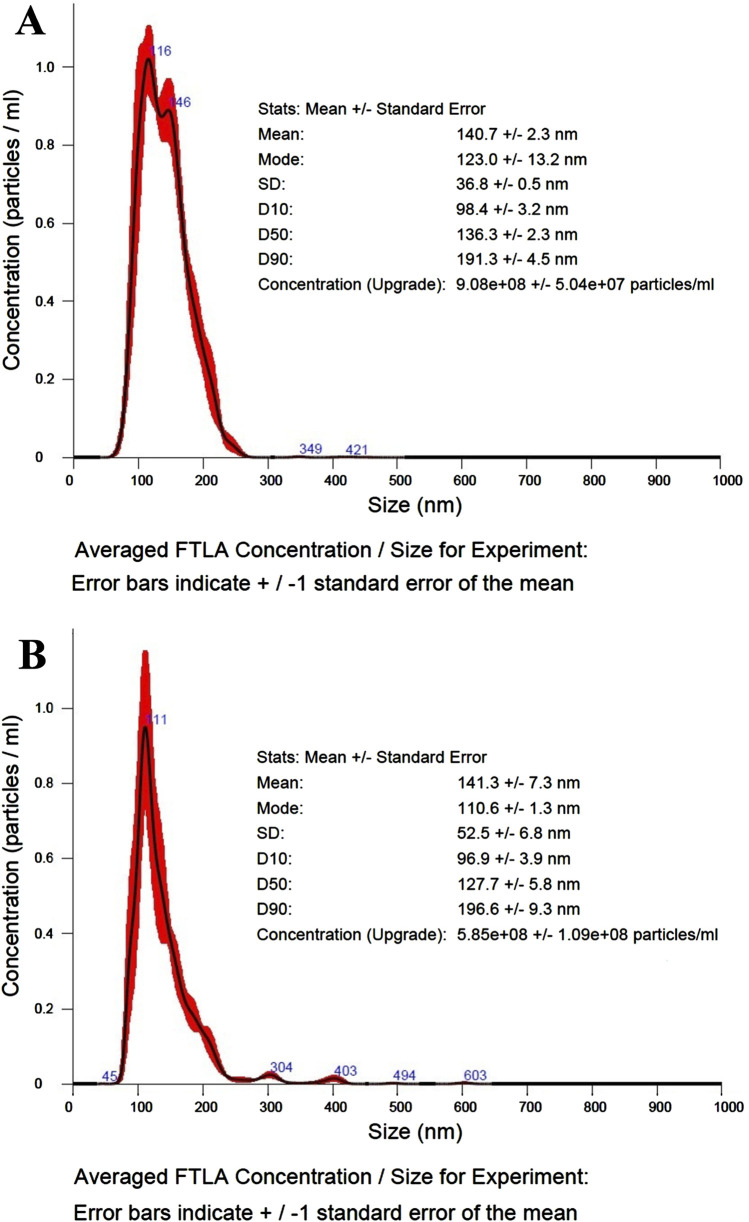
NTA analysis of control and recombinant OMVs. **(A)** NTA size/concentration distribution curve, average curve from 3 measurement of control OMVs (dilution 250-fold). **(B)** NTA size/concentration distribution curve, average curve from 3 measurement of ClyA-AR23 OMVs (dilution 100-fold).

The nano-tracking (NTA) analysis performed on both control and recombinant OMVs revealed no significant differences in either OMV size and distribution. As evident in Figure 4, both vesicle preparations showed an average monodisperse population with mode size values around 110–120 nm and mean size values of 140 nm. The primary evident difference between the preparations was vesicle concentration, with the control sample showing nearly 4-fold higher concentration compared to the recombinant preparation.

### 2.3 ClyA-AR23 protein identification by LC-MS/MS

ClyA-AR23 recombinant expression was verified by proteomic analysis, using both PD and Mascott softwares. The results showed a significant overexpression of ClyA protein in both recombinant cells and vesicles, which was confirmed by both software used.

More in detail, biostatistical analysis obtained with PD software revealed that the abundance of ClyA protein was almost 50 times higher in cell lysates and 12 times higher in OMVs obtained from cells transformed with pET22b (+)/ClyA-AR23 compared to controls (Proteome Discoverer data in Table 1). Consistently, normalized Mascot scores found in samples obtained from cells transformed with pET22b (+)/ClyA-AR23 were approximately 10 times higher compared to controls (Mascot data in Table 1).

**TABLE 1:** Proteome discoverer ratios and Mascot abundances of ClyA protein in proteomic analyses.

Proteome discoverer		
Samples ratio	Abundance ratio for ClyA	p-value
AR-23 lysates/Ctrl lysates	48.6	3.9*E-07
AR-23 OMVs/Ctrl OMVs	12.5	0.035

Mascot			
Samples	ClyA scores	Total scores	Normalized scores
Ctrl Cell lysates	354	295,967	1.2
Ctrl OMVs	69	65,609	1.1
AR-23 Cell lysates	3,761	350,105	10.7
AR-23 OMVs	601	44,653	13.5

**FIGURE 5:**
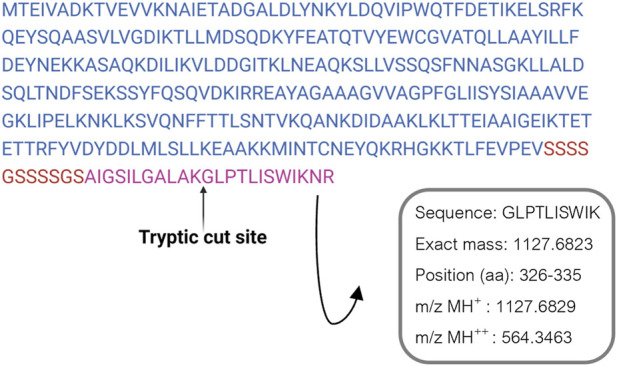
ClyA-AR23 recombinant protein sequence and its last tryptic peptide. The amminoacidic sequence, exact mass and *m/z* values of AR-23 tryptic peptide selected are shown.

**FIGURE 6:**
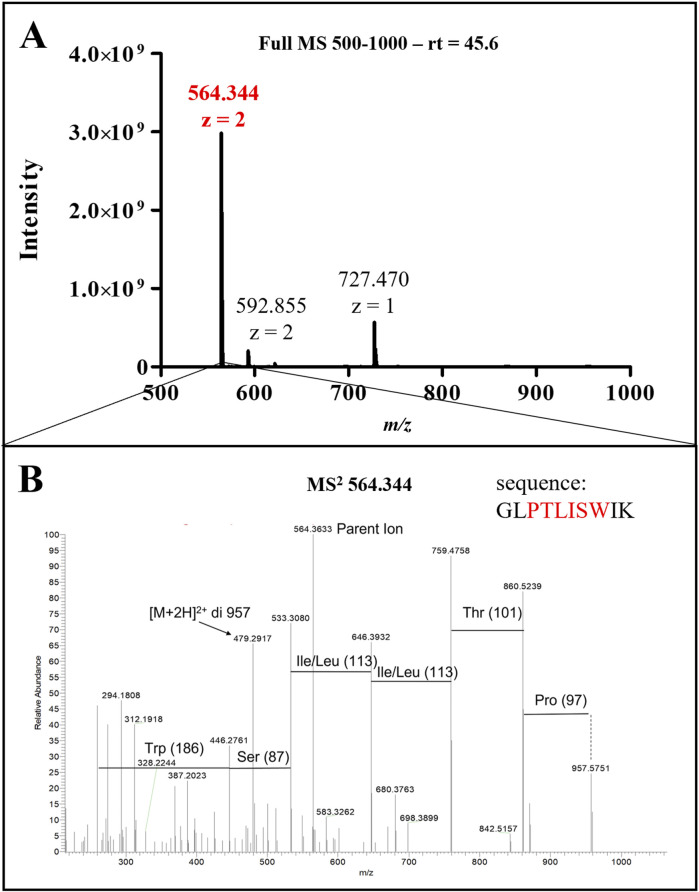
LC-MS/MS analysis of AR-23 tryptic peptide. **(A)** Full MS spectrum (500–1,000 m/z) of the peak at retention time 45.6 min. **(B)** MS^2^ spectrum of 564.344 ion. Sequence highlightd in red in this panel correspond to the presented MS^2^ signals. MS raw data, obtained from Xcalibur QualBrowser, were reported in Graphpad Prism for visualization.

ClyA enrichment in recombinant cell lysates and OMVs was in accordance with recombinant protein production, even though it did not confirm the presence of AR-23 peptide in our recombinant system. To this purpose, in fact, we performed an LC-MS/MS analysis to specifically identify the AR-23 peptide in samples through the identification of its specific tryptic peptide *m/z* signals. We identified tryptic peptides derived from our recombinant chimeric protein from its sequence, as shown in Figure 5. The last tryptic peptide of the protein contained part of the AR-23 sequence, making its *m/z* value useful for the specific identification of AR-23 in our samples, as it displayed an optimal mass range for the identification in a double charged state. The signal of the AR-23 tryptic peptide was validated by performing an LC-MS/MS analysis of the AR-23 standard peptide in solution, reduced, carboxy-ammido methylated and digested with trypsin. The selected AR-23 tryptic peptide showed a retention time of 45.6 min in our chromatographic conditions. Its MS and MS^2^ spectra are shown in Figure 6. A prevalent *m/z* signal of 564.344, corresponding to the double charged peptide, was identified at retention time (rt) of 45.6 min (Figure 6A), as confirmed by its isotopic pattern (data not shown). The aminoacidic sequence and identity of the peptide signal were confirmed by its fragmentation spectrum, shown in Figure 6B, which highlighted the expected amino acids sequence.

Following its identification, the 564.344 *m/z* signal was further used to assess the differential AR-23 presence in cell lysates and vesicle preparations. Cells and vesicles gel slices samples were analyzed with a 60-min linear gradient and the 35–45 kDa gel slices, corresponding to ClyA-AR23 molecular weight, were used to confirm the presence of the antiviral peptide.

**FIGURE 7:**
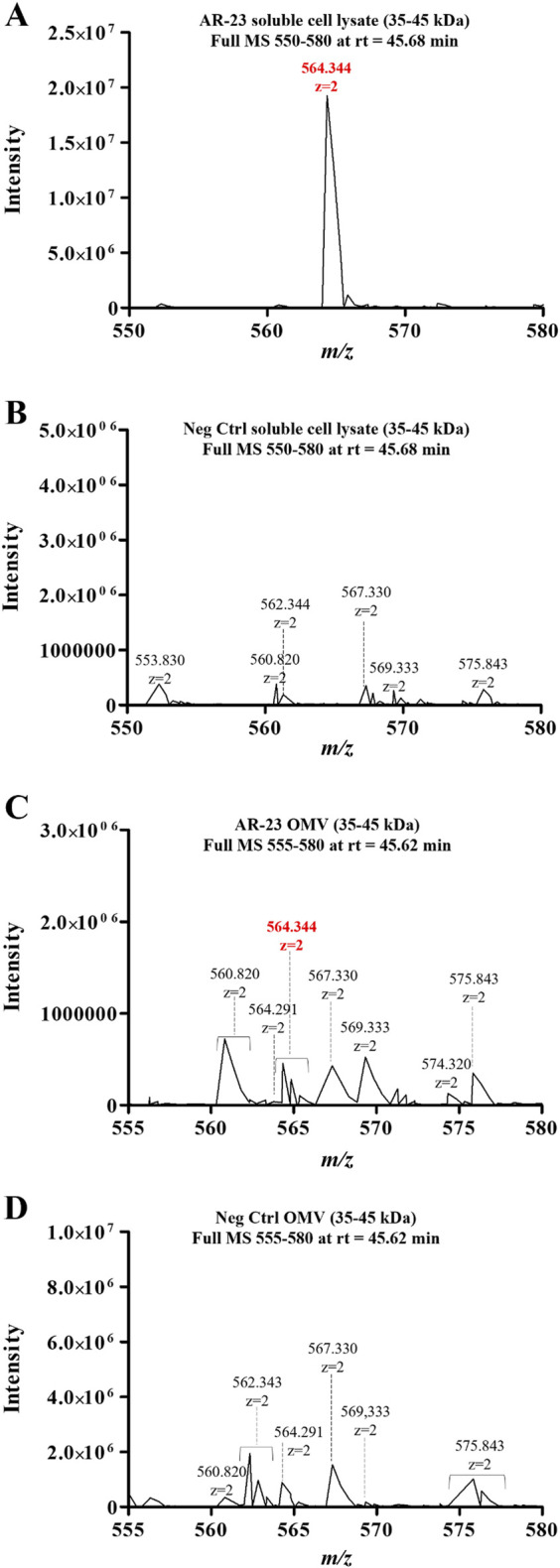
LC-MS/MS analysis of cell lysates and OMV fractions. **(A)** ClyA-AR23 cell lysates Full MS spectrum (*m/z*:550–580). **(B)** Neg Ctrl cell lysates Full MS spectrum (*m/z*:550–580). **(C)** ClyA-AR23 OMVs, Full MS spectrum (*m/z*:555–580). **(D)** Neg Ctrl OMVs, Full MS spectrum (*m/z*:555–580). 35–45 kDa gel fractions were analyzed for all samples and Full MS at rt of 45.6 min were extracted. MS raw data, obtained from Xcalibur QualBrowser, were reported in Graphpad Prism for visualization.

In Figure 7, the 564.344 ion full MS spectra extracted for the retention time of AR23 tryptic peptide (45.6 min) are reported. Panels A and B in Figure 7 show the data from soluble fractions of cell lysates of control and recombinant cells, respectively. The extracted mass spectra showed the presence of a double charged *m/z* signal of 564.344 only in recombinant cell lysates (Figure 7A). Similarly, panels C and D show the mass spectra of OMV samples, confirming the presence of the same expected *m/z* signal, exclusively in recombinant vesicles (Figure 7C).

However, in this latter case, the signals were less intense compared to cellular fractions. Nonetheless, both fractions displayed the presence of this differential signal in otherwise highly similar spectra. Moreover, the identity of the 564.344 *m/z* signal in cell lysates was validated using its MS^2^ spectrum, confirming the expected aminoacidic sequence (Supplementary Figure S2). Conversely, no fragmentation spectrum could be obtained from OMV samples, due to the low signal intensity, though its *m/z* and retention time matched the validated ones in cell lysates.

### 2.4 Recombinant expression of ClyA-his scaffold protein and evaluation of its surface exposure in *Escherichia coli* BL21 (DE3) cells and OMVs

**FIGURE 8:**
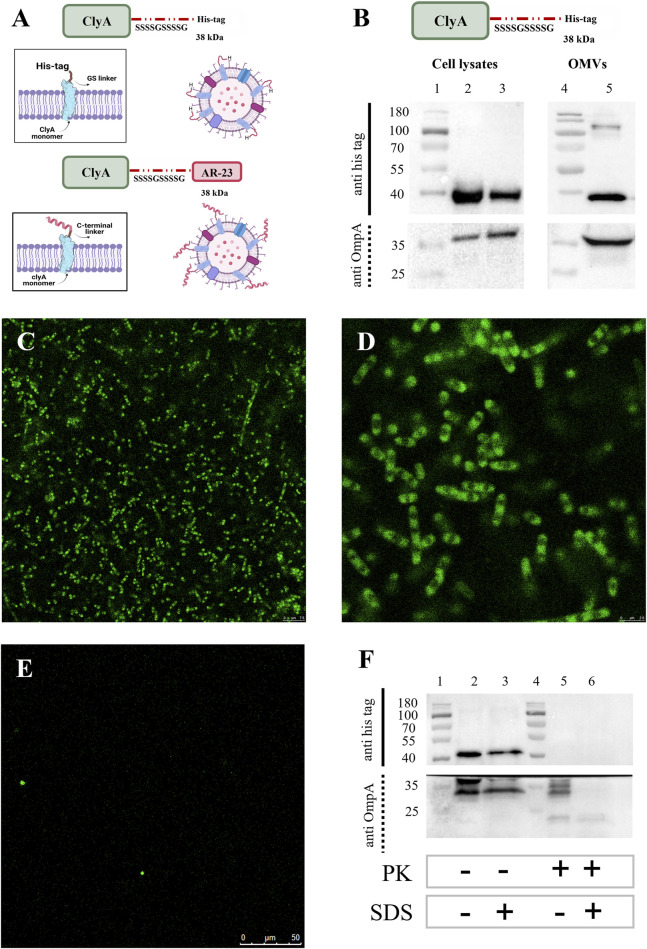
Expression and surface localization of ClyAhis scaffold protein. **(A)** ClyAhis and ClyA-AR23. **(B)** Western blot analysis of ClyAhis expression in *Escherichia coli* BL21 (DE3) cells. Lane 1 and 4: MW markers, lane 2: soluble fraction of cell lysates, lane 3: insoluble fraction of cell lysates, lane 5: OMVs. The upper panel sections show anti His tag antibody signals and the lower panels show the anti OmpA antibody signals. **(C,D)** Confocal microscopy images of *Escherichia coli* BL21 (DE3) cells expressing ClyAhis in immunofluorescence experiments. **(E)** Confocal microscopy images of *Escherichia coli* BL21 (DE3) negative control cells, transformed with pET22b (+) plasmid. **(F)** Western blot of Proteinase K limited proteolysis assay on ClyAhis OMVs. Lanes 1 and 4: MW markers, lane 2: untreated ClyAhis OMVs, lane 3: ClyAhis OMV in presence of 2% SDS, lane 5: ClyAhis OMVs in presence of 1 μg/mL PK, lane 6: ClyAhis OMVs in presence of 2% SDS and 1 μg/mL PK. The upper panel sections show anti his tag antibody signals and the lower panels show the anti OmpA antibody signals.

Given the lack of an antibody specific for ClyA or AR23 peptide, to give insight on the correct exposure of AR-23 peptide on the outer membrane extracellular side we decided to monitor instead the surface exposure using the ClyA recombinant scaffold protein fused with a C-terminal His tag. This latter was used in Western blot analysis using an anti-His tag antibody. This protein, here referred to as ClyA-his, served as a verification of the correct localization and surface exposure of the scaffold protein used to construct the ClyA-AR23 chimera, which differs from ClyA-his for the presence of the AR-23 peptide and absence of his tag at the C-terminus (Figure 8A).

ClyA-his was recombinantly expressed in *E. coli* BL21 (DE3) cells in the same conditions used for ClyA-AR23, and its presence in cell lysates and OMVs was screened by Western blot analysis, as reported in Figure 8B. An anti-OmpA antibody, directed towards the bacterial Outer membrane protein A, was used as control. ClyA-his C-terminus exposure towards the extracellular environment was verified in both cells and OMVs using two different strategies. The first consisted of demonstrating that ClyA-his was effectively exposed on the outer membrane of recombinant *E. coli* cells. To this purpose, we performed immunofluorescence analyses on bacteria after the expression of the recombinant protein. BL21 (DE3) cells transformed with pET22b (+) empty plasmid were used as a negative control. An anti-His tag antibody was used as the primary antibody for all recombinant cells, and a secondary antibody conjugated with Alexa-Fluor 488 fluorophore was used for detection. Stained bacteria were then spotted on slides and images were acquired by confocal microscopy. Representative images obtained for ClyA-his bacteria are shown in Panels C and D of Figure 8 and highlighted the presence of the fluorescent signal on the surface of recombinant bacteria. Conversely, no fluorescence was recorded in confocal microscopy images of negative control bacteria, as shown in Figure 8E, thus confirming the specificity of the signal. Furthermore, a limited proteolysis assay followed by Western blot analysis was carried out. ClyA-his-expressing OMVs were incubated with Proteinase K under controlled conditions (short incubation time and a low enzyme concentration) to obtain selective digestion only of proteins exposed on the vesicle surface, thus avoiding hydrolysis of proteins contained in the vesicle lumen or enveloped in the membrane. The actual digestion of the recombinant proteins was monitored by Western blot analysis, using the anti-His tag antibody. As a control, we used an antibody directed towards an OmpA epitope localized on the inside membrane leaflet, which should be protected from proteolysis. The same protocol was carried out on OMVs incubated in presence of 2% of sodium dodecyl sulphate (SDS), functioning as an anionic surfactant detergent and causing OMVs membrane de-structuration. Under such conditions, signals related to both his tag and OmpA should not be observed since membrane degradation would expose all protein components to PK degradation. Results are shown in Panel F of Figure 8. Western blot signals for ClyA-his were evident in the absence of PK regardless of the presence of 2% SDS (Lanes 2 and 3 in Figure 8F). When OMVs were incubated with PK alone, only the His tag signal failed to be identified, while that of OmpA remained evident (Lane 5 in Figure 8F). Finally, when both PK and SDS were incubated with OMVs, both signals disappeared or became faint (Lane 6 in Figure 8F). These results confirmed that the OmpA protein was exposed to proteolysis only when vesicles were disrupted. On the contrary, ClyA-his was digested in the presence of PK also in the absence of 2% SDS, thus suggesting that its C-terminus, where AR23 peptide was added in ClyA-AR23, was exposed on OMV surface and accessible to protease.

We used ClyA-his protein Western blot signal also to obtain an indication concerning the amount of recombinant protein packed in OMVs. To this end, a densitometric analysis was carried out on ClyA-his vesicles. A calibration curve was built in SDS-PAGE using different amounts of CD19-AlexaFluor 555 recombinant protein (ACROBiosystem), which contains a C-terminal His tag. Two different amounts of total proteins of ClyA-his OMVs were loaded on the same SDS-PAGE, and all samples were analysed by Western blot. The chimeric proteins signal intensities in OMVs were then plotted on the calibration curve obtained with CD19-AlexaFluor 555 recombinant protein (Supplementary Figure S3). A concentration of recombinant protein around 11 (+/−2) ng per μg of total proteins in vesicles was estimated, which represents approximately 1/90 of total OMV proteins. Although no data could be inferred about ClyA-AR23 enrichment in OMVs from this experiment, given that it is a different protein, this indication can still be considered indicative of ClyA display efficiency in our system.

### 2.5 Cell viability after OMVs exposure

**FIGURE 9:**
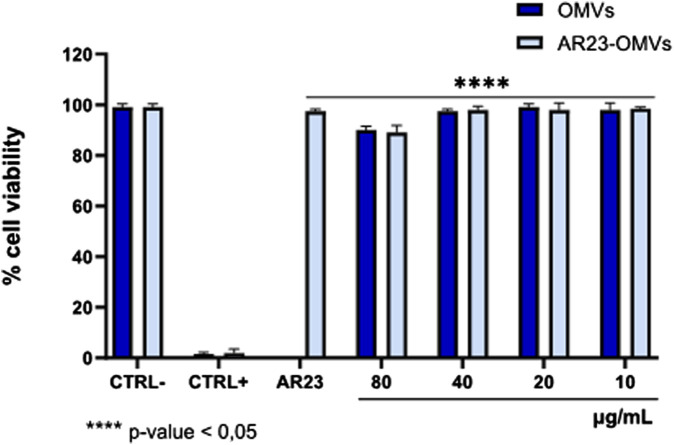
Cytotoxicity of ClyA-AR23 OMVs and OMVs purified from AR23-*Escherichia coli* and *Escherichia coli*. CTRL-consisted of cells exposed to the solvent used to resuspend the OMVs (1 × PBS), CTRL+ represented 100% DMSO and AR23 is associated with exposure to free peptide, at 6.25 μM. Data represent the mean ± SD of two independent experiments. The *p-value* <0.05 was considered significant.

The cytotoxicity of ClyA-AR23 OMVs and negative control OMVs were evaluated on VERO 76 cells by MTT assay (Figure 9). The cell monolayer exposed to vesicular samples for 24 h in the 10–80 μg/mL of proteins concentration range did not show significant alterations in cell viability. DMSO (CTRL+) caused 100% cell death while the solvent used to resuspend the OMVs (1X PBS) and the AR23 peptide control (6.25 μM) had no effect. At 80 μg/mL of protein concentration, both vesicular populations displayed approximately 11% toxicity, while at lower concentrations, the viability rate was comparable to the negative CTRL. The *p-values* obtained by statistical evaluation indicated that exposure to OMVs or OMV-AR23 did not significantly alter cell viability compared to CTRL−, confirming the absence of significant toxic effects at the cellular level.

### 2.6 Functional characterization of recombinant ClyA-AR23 OMVs antiviral activity

**FIGURE 10:**
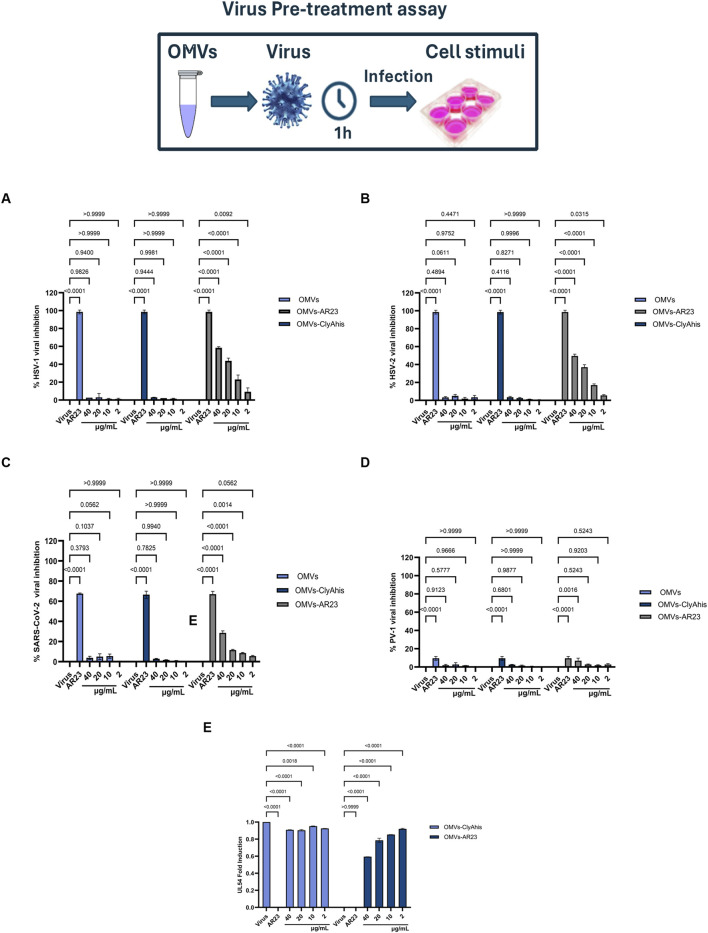
Antiviral activity of OMVs, ClyAhis OMVs and AR23-OMVs against enveloped and non-enveloped viruses. Virus pretreatment assay versus HSV-1 **(A)**, HSV-2 **(B)**, SARS-CoV-2 **(C)** and PV-1 **(D,E)** Analysis of HSV-1 UL54 expression levels after treatment with ClyAhis OMVs and AR23-OMVs. Data represent the mean ± SD of two independent experiments. The *p-value* <0.05 was considered significant.

The inhibitory activity of negative control, ClyA-his OMVs and ClyA-AR23 OMVs was investigated using a plaque reduction assay. Considering previous results, which highlighted an effective plaque reduction effect by exposing enveloped viruses to the AR-23 peptide (Chianese et al., 2022), the antiviral activity was evaluated in a virus pretreatment assay on viral strains HSV-1, HSV-2, SARS-COV2. PV-1 was tested as a non-enveloped virus. Results are shown in Figure 10. OMVs purified from the control *E. coli* BL21 (DE3) strain and ClyA-his OMVs did not compromise viral replication in the protein concentration range tested (total protein content of 2–40 μg/mL). Conversely, OMVs obtained from the engineered ClyA-AR23 *E. coli* strain were able to impair the replicative cycle of HSV-1. More in detail, an inhibition of 59.9% was recorded at the highest concentration tested (40 μg/mL), which decreased in a dose-dependent manner up to an inhibition of 22.5% observed at 10 μg/mL. Consistently with published data, AR-23 free peptide showed 100% inhibition in the extracellular phase at a concentration of 3.12 μM (Figure 10A). From the results obtained, it was possible to hypothesize that AR-23, exposed on OMVs, retained antiviral potential against HSV-1.

Since an accurate estimation of the effective amount of recombinant ClyA-AR23 proteins present on the surface of each OMV is not achievable, a straightforward comparison of the antiviral efficacy of the peptide in its free or immobilized form is not feasible. However, considering the total particles amount used in the assay and reported in Table 2, it can be inferred that -on a molar basis-at least 7,800 times less total particles of vesicles compared to purified AR-23 units were used. To explore the antiviral activity spectrum of ClyA-AR23 OMVs, further tests were performed against three other viruses: HSV-2, phylogenetically related to HSV-1; SARS-CoV-2, an enveloped RNA virus and PV-1, a non-enveloped RNA virus. Although HSV-2 shares a similar envelope architecture and entry glycoproteins to HSV-1, it differs substantially in terms of tropism, antigenicity, and replication kinetics. Therefore, HSV-2 serves as a relevant model to explore whether the antiviral activity of OMV-AR23 extends beyond HSV-1 to other herpesviruses. Indeed, OMV ClyA-AR23 demonstrated inhibitory effects against HSV-2, with a 50% and 35% reduction in viral infectivity at 40 and 20 μg/mL, respectively, confirming a dose-dependent antiviral activity moderately lower than what observed for HSV-1 (Figure 10B). In the case of SARS-CoV-2, OMV ClyA-AR23 achieved 29% inhibition at the highest vesicular dose tested, suggesting that the antiviral mechanism may involve interaction with envelope conserved in several enveloped viruses (Figure 10C). In contrast, no antiviral effect was observed against PV-1, indicating that OMV ClyA-AR23 was ineffective against non-enveloped viruses (Figure 10D). These results support the hypothesis that the antiviral activity of AR-23, when administered via OMV, is likely mediated by a direct interaction with viral envelope proteins, as previously proposed for the free peptide form. The lack of activity against a non-enveloped virus reinforces the idea that the viral envelope is the primary target of AR-23-mediated inhibition. To validate the findings from the plaque reduction assays, qPCR analysis was performed on HSV-1, the virus for which the highest inhibition was observed. The target of the analysis was the UL54 gene, which encodes the ICP27 protein, an essential factor that inhibits mRNA splicing in eukaryotic cells and facilitates the nuclear export of viral transcripts.

**TABLE 2:** Concentration and particles data used in the antiviral activity assays.

Samples	Concentration (M)	Particles/µL	Volume (µL)	Number of total particles
AR23 peptide	3.12*10^−6^	3.12*10^11^	250	7.80*10^13^
ClyA-AR23 OMVs	-	4.60*10^7^	250	1.15*10^10^
ClyAhis OMVs	-	5.00*10^7^	250	1.25*10^10^
Control OMVs	-	7.85*10^7^	250	1.96*10^10^

Particles per µl and volume used in the antiviral assay are reported. For OMVs, the average particles number measured by NTA, of the different preparations tested are reported, (3 for ClyA-AR23, and Control OMVs, and 2 for ClyAhis OMVs). The indicated values refer to the higher vesicles concentration content used, 40 µg/mL of total proteins.

For molecular analysis, the virus pretreatment assay was performed under the same experimental conditions described for the plaque reduction assay; RNA was then extracted from treated cells and retro-transcribed into cDNA. Real-time PCR results of cDNA samples showed that UL54 gene expression was inhibited, revealing a negative fold-induction of 0.57 and 0.79 at 40–20 μg/mL, respectively, after exposure to ClyA-A23-OMVs. On the other hand, viral gene expression following exposure to control OMVs from *E. coli* did not undergo any significant variation (Figure 10B).

## 3 Discussion

OMVs, which can be considered as phospholipid-based vesicles naturally produced by Gram-negative bacteria embedding important and modifiable molecular determinants of bacterial outer membrane, have recently emerged as a promising delivery platform to improve the bioavailability of several drugs. Among the advantages provided by OMVs as delivery systems, one of the most promising is the possibility to recombinantly express on their surface therapeutic-related oligo- and polypeptides. In this framework, several proteins can act as membrane-anchoring proteins for exposure such as the Outer membrane protein A, OmpA, an outer membrane protein widespread in Gram-negative bacteria, and Cytolysin A (ClyA), a transmembrane α-pore-forming toxin produced by some Enterobacteriaceae. Also, AMPs may be presented on the surface of OMVs by exposure to the outer leaflet of the vesicles through a variety of fusion proteins. Despite their well-described potential in biomedical applications, the main drawbacks of AMPs are related to their stability, lower immunogenic potential and the need for efficient delivery systems to improve their bioavailability and therapeutic efficacy (Wang et al., 2022). Expression of AMPs on OMV surface may increase their post-administration half-life and could be coupled with the decoration of OMV surface with proteins, providing their targeting to specific cells. In addition, OMVs intrinsically stimulate the immune system by delivering pathogen associated molecular patterns (PAMPs), thus activating innate and adaptive immunity.

To evaluate the implementation of *E. coli* OMVs as an antiviral agent presenting platform, we expressed an outer membrane fusion protein with ClyA bearing a C-terminal linker sequence of 11 serines and glycines, followed by AR-23 peptide. The efficacy and versatility of ClyA as a surface exposure tool on Gram-negative OMVs was extensively described in literature, along with the exposure of its C terminus on the vesicle surface (Kim et al., 2008; Huang et al., 2016; Wang et al., 2019).

To the best of our knowledge, the ClyA monomer has not been previously reported fused to an antimicrobial peptide. The recombinant expression of ClyA-AR23 protein was optimized and verified in *E. coli* cells and OMVs by LC-MS/MS analyses, even though the low abundance of AR-23 *m/z* signal in OMVs prevented us from obtaining a validation of its identity by fragmentation spectrum.

To validate ClyA C-terminus extracellular exposure, we exploited the recombinant ClyA protein fused to a C-terminal His tag for immunological revelation. Immunofluorescence and limited proteolysis experiments allowed to verify ClyA C-terminus exposure both in cells and vesicles surface. It must be taken into consideration that a key limitation in this approach is that ClyA-his is indeed a truncated form of recombinant ClyA-AR23. However, ClyA-his differs from ClyA-AR23 only for the presence of AR-23 peptide at the C-terminus in place of his tag. Therefore, it could be reasonably considered as a significant indication of our system feasibility for OMVs surface exposure.

The cytotoxicity of ClyA-AR23 OMVs and “naked” OMVs was assessed using the MTT assay on VERO-76 cells. At a concentration of 80 μg/mL, both vesicular populations showed approximately 11% toxicity, while lower concentrations had negligible effects. Consistently, it was recently reported that 50 μg of *Klebsiella pneumoniae* OMVs induced a 25% reduction in BEAS-2B cell viability (Dell'Annunziata et al., 2024). The results of this study provide valuable insights into the antiviral properties of AR23-complexed OMVs against HSV-1. The plaque reduction assay demonstrated that while *E. coli*-derived control OMVs showed no significant effect on viral replication in the tested concentration range (2–40 μg/mL), ClyA-AR23 OMVs did effectively impair the HSV-1 replication cycle in a dose-dependent manner. In particular, the highest tested concentration of 40 μg/mL resulted in 59.9% inhibition of viral replication, which gradually decreased to 22.5% at 10 μg/mL. These data suggested that OMVs could act as a delivery vehicle for AR23. These results are consistent with previous studies demonstrating the potent antiviral activity of AR-23 in the extracellular phase, where it achieved complete inhibition of HSV-1 at a concentration of 3.12 μM. The ability of OMVs to retain and transmit the inhibitory effects of AR23 suggests a potential application in targeted antiviral strategies, given also their limited cytotoxicity. Therefore, OMVs implemented as a delivery system may improve the bioavailability and controlled release of antimicrobial peptides such as AR23. To further validate these results, qPCR analysis of the HSV-1 UL54 gene was performed. The qPCR results confirmed the plaque reduction assay data, showing significant inhibition of viral gene expression following viral pretreatment with ClyA-AR23 OMVs. Fold-induction values of 0.57 and 0.79 were observed at 40 μg/mL and 20 μg/mL, respectively. In contrast, exposure to control OMVs did not alter viral gene expression, supporting the conclusion that AR23 is the primary antiviral agent in this system. Considering the results obtained against HSV-1, the investigation was expanded to evaluate whether the antiviral activity of OMVs-ClyA-AR23 also extended to other viruses with distinct structural and genomic features. Specifically, efficacy was assessed against HSV-2, a virus closely related to HSV-1 but differing in tissue tropism and antigenic profile. The data revealed a reduction in viral infectivity of 50% and 35% at concentrations of 40 and 20 μg/mL, indicating a dose-dependent antiviral effect, albeit less significant compared to HSV-1. Regarding SARS-CoV-2, OMVs carrying AR23 achieved a 29% inhibition at the highest viral concentration tested. These findings suggested that the antiviral mechanism may also involve interaction with envelope glycoproteins, potentially conserved among phylogenetically related viruses. In contrast, no antiviral activity was detected against PV-1, indicating that the envelope could be a target for AR23. Overall, these results support the hypothesis that the primary target of AR23, even when delivered via vesicles, resided in structural components of the viral envelope, such as lipids or glycoproteins.

Although the antiviral efficacy of functionalized OMVs is not directly comparable to that of the peptide alone, the total particle data of AR-23 peptide compared to total OMV particles used in the assay showed almost a 7,800-fold increase in terms of total antimicrobial agent used for AR-23 peptide. Even though we have limited insights regarding the total amount of ClyA-AR23 protein per vesicle, which would allow to compare directly the molar concentration of AR-23 used in the two conditions, we could infer that the 60% HSV-1 inhibition and 50% HSV-2 inhibition observed for 40 µg/mL of OMVs could be significantly down-estimated. Moreover, AR-23 peptide fused to ClyA could display differences in peptide orientation, and its availability to the viral envelope could be modified compared to the free peptide. Certainly, further studies are needed to better unravel the mechanism of action of these functionalized OMVs.

We acknowledge that a key limitation of this study consists in the lack of a quantitative evaluation of ClyA-AR23 protein in OMVs. An indirect indication was obtained for ClyA-his protein, through a densitometric analysis highlighting that around 11 ng of ClyA-his are present for 1 μg of OMVs total proteins. However, it cannot be considered representative of ClyA-AR23 expression and an alternative approach needs to be set up to obtain an accurate quantification of this recombinant protein.

In this research, our aim has been to set-up, recombinantly express and partially characterize in terms of antiviral efficiency a chimeric system bearing the AR23 peptide fused to a well-known membrane protein such as ClyA, used as a scaffold. Our results pave the way to future studies concerning the use of AR23-OMVs as antiviral agents, thus confirming that OMVs represent a promising delivery strategy that not only optimizes the stability and distribution of the peptide but also leverages immune modulation to improve therapeutic outcomes.

## 4 Materials and Methods

### 4.1 Generals

*Escherichia coli* BL21 (DE3) and DH5α strains were purchased from Sigma-Merck. The plasmid used is pET22 (b+), from Thermo-Fisher Scientific. Luria-Bertani medium (LB) is the medium used in all bacterial cultures and recombinant expressions. Bacterial cultures, plasmid purifications and transformations were performed according to Sambrook (2001). Bacteriological agar (Sigma-Merck) was used to prepare solid media at a final concentration of 15 g/L. All media were sterilized before use in the autoclave by performing a 20-min cycle at 121 °C. Ampicillin (Sigma-Merck) was used as a selection antibiotic for transformed strains at a final concentration of 100 μg/mL. IPTG (isopropyl β-D-1-thiogalactopyranoside) was obtained from Thermo-Fisher scientific.

The pET22b (+) expression vector was acquired from Sigma-Merck. Bacterial growth was monitored by measuring the optical density expressed as OD/mL at 600 nm (OD600). The QIAprep Spin Miniprep Kit was used for plasmid DNA purification, while QIAquick Gel Extraction Kit and QIAquick PCR Purification Kit, obtained from Qiagen, were used for PCR Clean ups and gel extraction processes.

The T4 DNA Ligase, along with other restriction enzymes and buffers for DNA manipulation, was obtained from New England Biolabs. Taq DNA Polymerase from Qiagen, along with its dNTPs and reaction buffers, was utilized for PCR amplification. Coding sequences were synthesized by Eurofins Genomics. Protein quantification was performed using the Pierce™ Detergent Compatible Bradford Assay (Thermo-Fisher Scientific), which was obtained from Thermo-Fisher Scientific.

Polyacrylamide gel electrophoresis was performed using standard techniques (He, 2011). SDS–PAGE was carried out with 12.5% Tris–glycine gels under reducing and denaturing conditions. Laemmli buffer (125 mM Tris HCl pH 6.8, 4% SDS, 20% glycerol, 0.004% bromophenol blue, 10% 2-mercaptoethanol) was added to the samples, which were boiled for 10 min at 100 °C. Running buffer was 1X Tris-glycine SDS buffer, pH 8.8 (0.3% Tris, 1.44% glycine, and 0.1% SDS). Electrophoresis was performed at 100 V constant voltage at RT; afterwards, the gel was incubated in a protein fixing solution (40% methanol, 5% acetic acid in water) for 15 min. Three washes with deionized water were performed for 10 min each, and gels were stained with Gel-Code Blue Stain (Thermo-Fisher Scientific) by incubating them under gentle shaking for 1 h at RT. Decoloration was obtained by washing gels in deionized water for 30 min. PageRuler™ Prestained Protein Ladder, 10–180 kDa (Thermo-Fisher Scientific) was used as a molecular weight marker.

### 4.2 Construction of pET22b (+)/ClyA-AR23 expression vector

The coding sequence for the ClyA monomer protein used in Huang et al. (2016), fused to a C-terminal linker of glycines and serines (GS linker), was designed and synthesized by Eurofins Genomics. This sequence, referred to as ClyA-GS, contains unique restriction sites, including *NdeI* upstream and a *XhoI* downstream. The sequence was cloned into pET22b (+) plasmid, in frame with a his tag, followed by a stop codon. This coding sequence is the one used to recombinantly express ClyAhis protein (in this case the plasmid is named pET22b (+)/ClyAhis). A *BamHI* unique restriction site was also inserted into the GS linker coding sequence. The ClyA-GS sequence cloned into the pET22b (+) vector will hence forth be referred to as pET22b (+)/ClyA-GS, and its sequence is shown in Supplementary File S1.

The coding sequence for AR-23 was designed and optimized for *E. coli* codon usage via the GENEius tool (Eurofins Genomics). The optimized sequence with a terminal stop codon was synthesized by Eurofins Genomics with a *BamHI* upstream and *XhoI* downstream unique restriction sites, and cloned in a pEX-A128 vector. The sequence of this vector, here referred to as pEX/AR-23, are shown in Supplementary File S2. Plasmids and cloning strategy are shown in Figure 1. The AR-23 coding sequence was first amplified from pEX/AR-23 plasmid using the pEX-For and pEX-Rev priming sites in the pEX plasmid (in red in Supplementary File S2). Oligonucleotides for PCR amplification were purchased from Eurofins Genomics. The amplified fragment underwent a liquid clean up procedure. Subsequently, both the AR-23 PCR product and pET22b (+)/ClyA-GS were digested using *BamHI* and *XhoI* enzymes (NEB). Briefly, 1.5 μg of total DNA of pET22b (+)/ClyA-GS and AR-23 PCR product were separately incubated with 20 total units of both enzymes for 3 h at 37 °C, following manufacturer’s procedure (NEB). Then, pET22b (+)/ClyA-GS digestion products were loaded on 1% agarose gel and the fragment of interest, containing pET22b (+) and ClyA coding sequence, was excised and purified with QIAquick Gel Extraction Kit (Qiagen).

Conversely, the AR-23 PCR product, after endonucleases digestion, underwent a liquid clean up procedure using the QIAquick PCR Purification Kit (Qiagen). DNA fragments of both the plasmid and PCR product were quantified using a Nano-Photometer (N80 - Euroclone). Subsequently, the DNA fragments were ligated at a 1:3 M ratio (vector: fragment) with T4 DNA Ligase (NEB) and incubated overnight at 16 °C. Ligation products were used to transform the *E. coli* DH5α strain. Selected colonies were inoculated overnight in 10 mL of LB medium with 100 μg/mL ampicillin. Recombinant plasmids were purified and sequenced by Eurofins Genomics, confirming that the correct coding sequence was obtained. The resulting construct, coding for the chimeric protein ClyA-AR23 is shown in Supplementary File S3. The pET22b (+) vector was used as an expression vector in *E. coli* BL21DE (3) cells for subsequent experiments.

### 4.3 ClyAhis and ClyA-AR23 recombinant expression

Transformation of *E. coli* BL21DE (3) competent cells with either pET22b (+)/ClyAhis or pET22b (+)/ClyA-AR23 was performed according to Sambrook (2001). Transformed bacteria were grown from the transformation plate and inoculated in 10 mL of LB broth, containing 100 μg/mL of ampicillin (LB-amp). Pre-inocula were incubated at 37 °C with constant shaking until approximately 0.5 OD_600_. Protein expression (ClyAhis and ClyA-AR23) was induced with 0.5 mM IPTG, and growth was continued for 3 h at 37 °C. Cells were harvested by centrifugation (5,524 × g for 15 min at 4 °C) and stored at −20 °C until needed. *Escherichia coli* BL21 (DE3) competent cells transformed with pET22b (+) empty plasmid were grown and processed identically as a negative control in subsequent experiments.

### 4.4 OMVs isolation and purification

The exhausted media obtained from *E. coli* BL21 (DE3), either expressing ClyAhis, ClyA-AR23 or transformed with an empty vector (these latter are named in this manuscript as “control” cells) were collected after the 3-h induction period via centrifugation, as described previously. Media used for OMVs purification were filtered through 0.45 µm PVDF membranes (Sigma-Millipore) and concentrated up to 40 mL using an Amicon ultrafiltration device with a 30 kDa cut-off (Millipore YM30 membrane). The concentrated media were further filtered through 0.22 µm membranes (Sigma-Millipore), ultracentrifuged at 170,000 × *g* for 2.5 h at 4 °C, and the pellets resuspended in 1X Phosphate-buffered saline (PBS) (PanReac AppliChem). After a second ultracentrifugation under the same conditions, the PBS supernatant was discarded, and the OMV-containing pellets were resuspended in 300–500 µL of 1X PBS and stored at −20 °C until further use.

### 4.5 OMVs nano-tracking analysis (NTA)

The size, distribution and concentration of both control and recombinant OMVs were determined using Nanoparticle Tracking Analysis (NTA) with a NanoSight NS300 instrument (Malvern Instruments, Malvern, United Kingdom) equipped with a green laser and a sCMOS camera. Samples were diluted in MilliQ water, filtered with a 0.22 µm syringe filter and injected into the laser chamber. Vesicles were diluted to obtain several particles per frame comprised between 20 and 120. Data acquisition was performed at a syringe speed of 30 infusion rate units with camera levels set between 12 and 15 and detection threshold set at 5. Three videos of 60 s were recorded for each measurement at room temperature. Subsequently, videos were analysed using NanoSight NTA 3.4 software to perform tracking analysis. All measurements were performed in triplicate.

### 4.6 Proteomic analysis of native and recombinant cell lysates and OMVs

The cell paste of native and recombinant cells was resuspended in 25 mM MOPS (3-morpholino-1-propanesulfonic acid) pH 6.9 to a final concentration of approximately 70–80 OD_600_ with 1 mM PMSF (phenyl methyl sulfonyl fluoride) as a protease inhibitor. Cells were disrupted by sonication (30 times for a 30 s ON cycle, on ice, with 10% amplitude). Cell debris were removed by centrifugation at 22,100 g for 20 min at 4 °C. The supernatant was collected as the soluble fraction, filtered through a 0.45 μm PVDF Millipore membrane and stored at −80 °C. An aliquot of the pellet was resuspended in Laemmli buffer as the insoluble fraction. Both soluble fractions of cell lysates and OMVs were analysed for protein concentration via the Bradford assay. Five micrograms of OMVs total proteins and 15 µg of lysates (soluble and insoluble fractions) were loaded onto a 12.5% polyacrylamide gel and SDS-PAGE was performed as outlined in paragraph 2.1. After electrophoresis, each lane of SDS-PAGE gel was cut into 10 pieces, which underwent trypsin in-gel digestion. Briefly, gel bands were washed, dehydrated with acetonitrile (ACN) and proteins in the gel were subjected to cysteine reduction using dithiothreitol (DTT) and carboxy-amido methylation using iodoacetamide (IAA). Finally, proteins underwent a trypsin digestion treatment in the presence of 25 mM ammonium bicarbonate (AMBIC) buffer with 7.5 ng/μL of Trypsin (Trypsin/Lys-C Mix, Mass Spec Grade, Promega) overnight at 37 °C. The peptide mixtures obtained were eluted from gel, vacuum dried, resuspended in 1% formic acid in water and analysed on an electrospray (ESI) Orbitrap Q-Exactive Mass Spectrometer (Thermo-Fisher Scientific) coupled with a nanoUltimate300 UHPLC system (Thermo-Fisher Scientific). Peptide separation was achieved using a capillary EASY-Spray PepMap column (0.075 mmÅ∼ 50 mm, 2 μm, Thermo Fisher Scientific) with aqueous 0.1% formic acid (A) and ACN containing 0.1% formic acid (B) as mobile phases. A linear gradient from 3% to 40% of B in 60 min and a 300 nL/min flow rate was used. Mass spectra were acquired over an *m/z* range from 375 to 1,500. MS and MS/MS data were analysed using Mascot software (v2.5, Matrix Science) with the non-redundant Data Bank UniprotKB/Swiss-Prot (Release 2023_03) database. Parameters included: *i* trypsin cleavage; *ii* carboxy-amido methylation of cysteines as a fixed modification and methionine oxidation as a variable modification; a maximum of two missed cleavages; *iii* false discovery rate (FDR), calculated by searching the decoy database, 0.05. Proteome Discoverer 2.4.1.15 software analyzed data using the UniprotKB *E. coli* database. Proteomic analyses were performed in duplicate on different lysates and vesicle preparations. Gene ontology analysis was done using UniProt Knowledgebase and STRING softwares. Data from Mascot software were used to calculate the Normalized Mascot scores using the Equation 1:

$$Normalized\;Mascot\;score=\left(\frac{ClyA\;score\;in\;sample}{total\;score\;of\;the\;sample}\right)*1000$$ (1)

### 4.7 ClyAhis Western blot assay

Proteins were separated on a polyacrylamide gel and transferred onto a PVDF membrane (Thermo-Fisher scientific). Transfer was carried out in the 1X Tris-glycine transfer buffer in the presence of 20% Methanol (MeOH), with a constant Voltage of 100 V at 4 °C for 1 h. When needed, after blotting, the membrane was cut horizontally between 35 and 40 kDa marker band; obtaining two membranes. For both, a blocking procedure was carried out, incubating the membranes in 1X PBS solution at pH 7 with 0.1% Tween detergent and 5% powdered milk (T-PBS 5% Milk), with gentle shaking overnight at 4 °C, to saturate non-specific binding sites. Membranes were then incubated in the presence of anti his tag antibody (over 35 kDa membranes) and anti-OmpA antibodies (down 35 kDa membranes), at a dilution between 1:5,000 and 1:10,000 for anti his tag and 1:25,000 for anti-OmpA in T-PBS 5% Milk for 1 h with gentle shaking at RT to promote hybridization (Rabbit Anti-HisTag Polyclonal Antibody–Elabscience or Rabbit Anti-OmpA Monoclonal Antibody–Antibody Research Corporation). Afterwards, membranes were washed three times with 1X T-PBS with shaking at RT for 10 min, to remove the excess of unbound antibodies. Membranes were hybridized with a secondary antibody conjugated to horseradish peroxidase (peroxidase/HRP-conjugated Goat Anti-Rabbit IgG (H+L) – Elabscience), at a 1:5,000 dilution in T-PBS, 5% Milk for 1 h under gentle shaking at RT. Membranes were washed again three times with T-PBS under shaking at RT for 10 min, to remove the excess secondary antibody. After incubation of chemiluminescent substrate of horseradish peroxidase (Excellent Chemiluminescent Substrate–ECL–Elabscience), the signal was detected using a UV transilluminator (ChemiDoc XRS–BioRad).

### 4.8 *Escherichia coli* BL21 (DE3) immunofluorescence and confocal microscopy

Cell pellets of BL21 (DE3) cells transformed with either pET-ClyAhis or pET22b (+) plasmids were collected after recombinant expression and resuspended in 1X PBS at a cell density of 4 OD600/mL, and 1 mL of the sample was used for each analysis. Samples were washed twice in 1X PBS at 1,200 × *g* for 3 min. Pellets were then resuspended in 1 mL of 4% paraformaldehyde in 1X PBS for cell fixation and incubated at 4 °C for 20 min with gentle agitation. Samples were then centrifuged at 1,200 × *g* for 3 min; supernatants were discarded, and pellets were washed three times with 1 mL of 1X PBS at 1,200 × *g* for 3 min. Then a blocking procedure was carried out, incubating bacterial cells in 1 mL of 1X PBS containing 1% BSA and kept under gentle agitation at RT for 1 h. Samples were then centrifuged at 1,200 × *g* for 3 min, and 1 mL of primary anti-His tag antibody (Rabbit Anti-HisTag Polyclonal Antibody–Elabscience) diluted 1:1,000 in PBS-1% BSA was added and incubated at RT for 1 h in gentle shaking. After incubation, samples were centrifuged, and pellets were washed twice with 1X PBS. Pellets were resuspended in 1 mL of secondary antibody conjugated with fluorophore Alexa-Fluor 488 (AlexaFluor 488-conjugated Goat anti-rabbit IgG (H+L)) diluted 1:500 in PBS-1% BSA and incubated at RT in the dark for 1 h with gentle agitation. After incubation, samples were centrifuged at 1,200 × *g* for 3 min and pellets were resuspended in 1 mL of 1X PBS and washed twice. Pellets were finally resuspended in 100 µL of 1X PBS to obtain a 40 OD600/mL of cell density. Twenty microliters of the samples were put on the Superfrost® microscope slides, closed with coverslips and fixed using 50% glycerol in 1X PBS. Images were acquired using a laser scanning confocal microscope TCS SP5 (Leica MicroSystems, Mannheim, Germany) equipped with a plan 63X, oil immersion objective lens.

### 4.9 Proteinase K limited proteolysis assay on OMVs

ClyAhis OMVs aliquots corresponding to 5 µg of total proteins were used for each digestion. Proteinase K limited proteolysis was carried out under various conditions, in the presence of 25 mM Tris HCl working buffer at pH 7.5 containing 10 mM CaCl2, 50 ng total of Proteinase K (PK) and, where required, 2% SDS, in a total volume of 50 µL. Mixtures were incubated for 15 min at 37 °C. Then, Laemmli buffer was added to the samples, which were boiled for 10 min at 100 °C. Samples were finally loaded on SDS-PAGE gels to be analysed by Western blot analysis as outlined in paragraph 4.7.

### 4.10 ClyAhis densitometric analysis in OMVs

A calibration curve was built in SDS-PAGE using different amounts (25, 50, 100 and 200 ng) of CD19-AlexaFluor 555 recombinant protein (ACROBiosystem), which contains a C-terminal His tag. 5 and 10 µg of total proteins of ClyAhis OMVs were loaded on the same SDS-PAGE, and all samples were analysed by Western blot using a Rabbit Anti-HisTag Polyclonal Antibody–Elabscience diluted 5,000-fold as primary antibody, and peroxidase/HRP-conjugated Goat Anti-Rabbit IgG (H+L) – Elabscience, diluted 5,000 fold as secondary antibody, as detailed above. The CD19-AlexaFluor 555 and ClyAhis signal were acquired using a UV transilluminator (ChemiDoc XRS–BioRad). Signal intensities in OMVs were then plotted on the calibration curve obtained with CD19-AlexaFluor 555 recombinant protein.

### 4.11 Cell line and viral strains

The cell line derived from the renal epithelium of the African green monkey (*Cercopithecus aethiops*, VERO-76) was purchased from the American Type Culture Collection (ATCC Manassas, Virginia, United States). For propagation, cells were cultured in Dulbecco’s Modified Eagle Medium (DMEM; Gibco; Thermo Fisher Scientific, Waltham, MA, United States) with 4.5 g/L glucose, 2 mM L-glutamine, 100 IU/mL penicillin-streptomycin solution and supplemented with 10% fetal bovine serum (FBS; Gibco; Thermo Fisher Scientific, Waltham, MA, United States) in a humidified atmosphere with 5% CO_2_ at 37 °C. Viruses used to evaluate the antiviral activity of OMVs included: HSV-1 strain SC16, containing a lacZ gene under the control of the cytomegalovirus IE-1 promoter for the expression of β-galactosidase; HSV-2 strain VR-1779; PV-1 strain VR-1562; and the SARS-CoV-2 strain VR-PV10734, kindly provided by the National Institute for Infectious Diseases “Lazzaro Spallanzani” in Rome, Italy.

### 4.12 Cytotoxicity of *Escherichia coli* OMVs

Before antiviral efficacy, the cytotoxicity of OMVs exposed to the VERO-76 cell line was determined using the 3-(4,5-dimethylthiazol-2-yl)-2,5-diphenyltetrazolium bromide (MTT) assay. Cells were seeded at a density of 2 × 104 cells/well in 96-well microtiter plates and incubated for 24 h at 37 °C in a humidified atmosphere with 5% CO2. Then, cells were exposed to OMVs obtained from ClyA-AR23 *E. coli* and control *E. coli* in the 80–10 μg/mL concentration range, for 24 h. Cells treated with 100% DMSO constituted positive control (CTRL+), samples exposed to the solvent used to dissolve and resuspend OMVs (1 × PBS) corresponded to the negative control (CTRL−) and cells incubated with AR23 peptide (6.25 μM) represented the peptide control. After treatment, 100 μL of MTT solution (Sigma-Aldrich, St. Louis, MO, United States) (0.3 mg/mL) was added to each well for 3 h at 37 °C. The formazan crystals formed were solubilized by adding 100 μL of DMSO (100%) for 10 min under vigorous orbital shaking. The cell viability rate was recorded by measuring the absorbance at 570 nm with a microplate reader (Tecan, Männedorf, Swiss), according to Equation 2:

$$\%\;cell\;viability=\frac{100*\left(OD\;570\;nm\;test\;sample\right)}{OD\;570\;nm\;CTR-sample}$$ (2)

### 4.13 *Escherichia coli* OMVs antiviral activity

For the antiviral activity of the engineered AR23 peptide in *E. coli-*OMVs, a virus pre-treatment assay was performed. VERO-76 cells were seeded at a density of 2 × 10^5^ cells/well in a 12-well plate and incubated overnight at 37 °C with 5% CO_2_. Then, OMVs derived from either AR23-*E. coli* or *E. coli* were diluted in 1 × PBS at a concentration range 2–40 μg/mL. Each preparation was pre-incubated with the viral suspension at 1 × 10^4^ plaque-forming units (PFU)/mL in DMEM without FBS for 1 h at 37 °C. The mixture was then diluted 1:10 in DMEM without FBS, and the virus at a multiplicity of infection (MOI) of 0.01 infected the cell monolayer for 1 h at 37 °C. After adsorption, cells were washed with citrate buffer (pH 3) (Sigma-Aldrich, St. Louis, MO, United States) to remove unpenetrated viral particles, and the cell monolayer was covered with DMEM supplemented with 10% FBS and supplemented with 5% carboxymethylcellulose (CMC) (Sigma-Aldrich, St. Louis, MO, United States). In agreement with previously published results (Chianese et al., 2023), the AR-23 peptide (3.12 μM) was used as CTRL+, while cells exposed to 1 × PBS represented the CTRL-. Infected cells were incubated for 48 h, and plaque counting was performed by fixing cells with 4% formaldehyde (Sigma-Aldrich, St. Louis, MO, United States), then stained with 0.5% crystal violet (Sigma-Aldrich, St. Louis, MO, United States). Viral inhibition rate was recorded from the plaque count and calculated according to Equation 3:

$$\%\;viral\;inhibition=100-\left[100*\left(\frac{plaques\;counted\;in\;test\;sample}{plaques\;counted\;in\;CTR-sample}\right)\right]$$ (3)

### 4.14 qPCR of HSV-1 gene viral transcript UL54

Plaque reduction assay was confirmed by quantitative polymerase chain reaction (qPCR) on UL54 gene viral transcript. The virus pretreatment test was performed as described above; after incubation, harvested cells were subjected to total RNA extraction with TRIzol (Thermo Fisher, Waltham, MA, United States). Then, 1 μg of RNA was reverse transcribed in cDNA following the instructions of the SensiFAST™ cDNA Synthesis Kit (Meridian Bioscience, Washington, DC, United States) and subjected to molecular amplification. The qPCR was performed in the Thermal Cycler UNO96 (VWR International, Pennsylvania, United States) using 1 μM of primer, 1X BrightGreen 2X qPCR MasterMix (abm, San Francisco, CA, United States) and 100 ng of cDNA. The amplification program consisted of denaturation at 95 °C for 15 s, annealing at 60 °C for 20 s and extension at 72 °C for 15 s (40 cycles). UL54 expression was assessed using the primers forward: 5′-TGGCGGACATTAAGGACATTG-3′ and reverse 3′-TGGCCGTCAACTCGCAG-5′. Values for the target threshold cycle (Ct) were normalized to the housekeeping gene glyceraldehyde 3-phosphate dehydrogenase (GAPDH), using the primers forward: 5′-CCTTTCATTGAGCTCCAT-3′ and reverse: 3′-CGTACATGGGAGCCGTC-5′.

### 4.15 Statistical analyses

The cytotoxicity and antiviral activity tests were performed in biological and technical duplicates and reported as mean ± standard deviation (SD). The significance of the differences of the samples exposed to OMVs with CTRL− was evaluated by one-way analysis of variance (ANOVA) associated with Dunnett’s test, using the Graph Pad Prism 9.0 software (San Diego, CA, United States). The results with a p-value <0.05 were considered statistically significant.

Statistical analyses of proteomic analyses were performed using Proteome Discoverer software with a two-sided unpaired t-test. Proteins were defined as differentially expressed when the p-value of their ratio across samples was <0.05.

## Data Availability

The mass spectrometry proteomics datasets generated for this study can be found in the ProteomeXchange Consortium via the “Proteomics Identification Database” PRIDE (https://www.ebi.ac.uk/pride/) partner repository with the dataset identifier: PXD061844. All other data are available within the article and its Supplementary Material.
