# Supplementary material for: Outer membrane vesicles from Escherichia coli as a presentation platform for AR-23 antiviral peptide
Source: Front Mol Biosci. 2025 Oct 3;12:1607578. doi: 10.3389/fmolb.2025.1607578 (PMC12531040; doi:10.3389/fmolb.2025.1607578)
Supplement: Supplementary file 1 [file Supplementaryfile1.docx]

Supplementary Material

# Supplementary Data

**S1 - ClyA-GS coding sequence in pET22b(+)**

ClyA-GS codying sequence cloned in pET22b(+). Restriction sites for *NdeI*, *BamHI*, *XhoI* are underlined. The ClyA codying sequence is highlighted in bold.

CAT**ATGACAGAGATTGTAGCAGATAAGACAGTCGAAGTGGTGAAGAATGCGATTGAAACCGCAGATGGAGCCCTGGATCTGTACAACAAGTACCTGGATCAGGTTATTCCCTGGCAAACGTTCGACGAAACCATCAAAGAGTTAAGCAGGTTCAAACAGGAATACTCTCAAGCAGCTTCGGTGCTTGTAGGCGATATTAAAACGTTATTGATGGACAGCCAGGACAAGTACTTCGAAGCCACACAGACGGTGTATGAATGGTGTGGAGTTGCTACCCAGCTATTAGCGGCCTATATCTTGTTGTTTGATGAGTACAATGAGAAGAAAGCTTCAGCCCAGAAAGACATCCTGATTAAAGTCCTGGATGACGGTATCACCAAACTGAATGAGGCACAAAAGTCTCTGTTAGTATCGAGCCAATCCTTCAACAATGCCAGCGGCAAACTTTTGGCGTTGGATAGTCAACTGACCAATGATTTCTCTGAGAAATCGAGCTACTTTCAGAGCCAAGTTGACAAAATTCGTCGTGAAGCGTATGCAGGTGCAGCTGCTGGTGTTGTAGCAGGACCATTTGGCCTCATTATCTCCTACAGCATTGCCGCTGCAGTTGTGGAAGGCAAGCTGATTCCTGAGCTGAAGAACAAGCTGAAAAGCGTTCAGAACTTCTTCACTACCCTTTCCAATACGGTGAAACAGGCGAATAAAGACATAGATGCTGCAAAGCTGAAACTTACGACTGAAATTGCTGCGATTGGGGAAATCAAAACCGAAACCGAAACTACTCGTTTCTATGTCGATTATGATGACTTGATGCTCAGTCTCCTCAAGGAAGCAGCGAAGAAGATGATAAACACCTGCAACGAATACCAGAAAAGGCATGGAAAGAAAACTCTATTTGAAGTTCCGGAAGTGTC**CTCCAGTTCCGGTTCTAGCAGTTCAGGATCCTCCTCAAGTGGAAGCTCCTCTTCTGGGAGCTCGTCATCAGGTAGCAGTAGCAGTGGATCTTCGTCAAGTGGGTCAAGTAGTAGCGGGTCGAGCAGTTCTGGCAGCTCATCTTCGGGTGGGTCGACCCTCGAGCACCACCACCACCACCACTGA

**S2 - AR-23 coding sequence in pEX-A128**

AR-23 codying sequence. Restriction sites for *BamHI* and *XhoI* are underlined. The AR-23 codying sequence is highlighted in bold and pEX-For, pEX-Rev priming sites are shown in red.

GGAGCAGACAAGCCCGTCAGGGCGCGTCAGCGGGTGTTGGCGGGTGTCGGGGCTGGCTTAACTATGCGGCATCAGAGCAGATTGTACTGAGAGAAAGGCAATTGGGTACCGAGCTCGCGGCCGCAAGCGTCGACCGGATCC**GCGATCGGCAGCATTCTGGGTGCCCTGGCGAAAGGCCTGCCGACCTTGATTTCGTGGATCAAAAACCGC**TAACTCGAGACCTGCTTTTGCTCGCTTGGATCCGAATTCAAAGGTGAAATTGTTATCCGCTCACAATTCCACACAACATACGAGCCGGAAGCATAAAGTGTAAAGCCTG

**S3 - ClyA-AR23 coding sequence obtained in pET22b(+).**

ClyA-AR23 codying sequence cloned in pET22b(+). Restriction sites for *NdeI*, *BamHI*, *XhoI* are underlined. The ClyA codying sequence is highlighted in bold and AR-23 codying sequence in red. GS linker sequence is in the middle.

CAT**ATGACAGAGATTGTAGCAGATAAGACAGTCGAAGTGGTGAAGAATGCGATTGAAACCGCAGATGGAGCCCTGGATCTGTACAACAAGTACCTGGATCAGGTTATTCCCTGGCAAACGTTCGACGAAACCATCAAAGAGTTAAGCAGGTTCAAACAGGAATACTCTCAAGCAGCTTCGGTGCTTGTAGGCGATATTAAAACGTTATTGATGGACAGCCAGGACAAGTACTTCGAAGCCACACAGACGGTGTATGAATGGTGTGGAGTTGCTACCCAGCTATTAGCGGCCTATATCTTGTTGTTTGATGAGTACAATGAGAAGAAAGCTTCAGCCCAGAAAGACATCCTGATTAAAGTCCTGGATGACGGTATCACCAAACTGAATGAGGCACAAAAGTCTCTGTTAGTATCGAGCCAATCCTTCAACAATGCCAGCGGCAAACTTTTGGCGTTGGATAGTCAACTGACCAATGATTTCTCTGAGAAATCGAGCTACTTTCAGAGCCAAGTTGACAAAATTCGTCGTGAAGCGTATGCAGGTGCAGCTGCTGGTGTTGTAGCAGGACCATTTGGCCTCATTATCTCCTACAGCATTGCCGCTGCAGTTGTGGAAGGCAAGCTGATTCCTGAGCTGAAGAACAAGCTGAAAAGCGTTCAGAACTTCTTCACTACCCTTTCCAATACGGTGAAACAGGCGAATAAAGACATAGATGCTGCAAAGCTGAAACTTACGACTGAAATTGCTGCGATTGGGGAAATCAAAACCGAAACCGAAACTACTCGTTTCTATGTCGATTATGATGACTTGATGCTCAGTCTCCTCAAGGAAGCAGCGAAGAAGATGATAAACACCTGCAACGAATACCAGAAAAGGCATGGAAAGAAAACTCTATTTGAAGTTCCGGAAGTGTC**CTCCAGTTCCGGTTCTAGCAGTTCAGGATCCGCGATCGGCAGCATTCTGGGTGCCCTGGCGAAAGGCCTGCCGACCTTGATTTCGTGGATCAAAAACCGCTAACTCGAG

# Supplementary Figures

**Supplementary Figure 1**


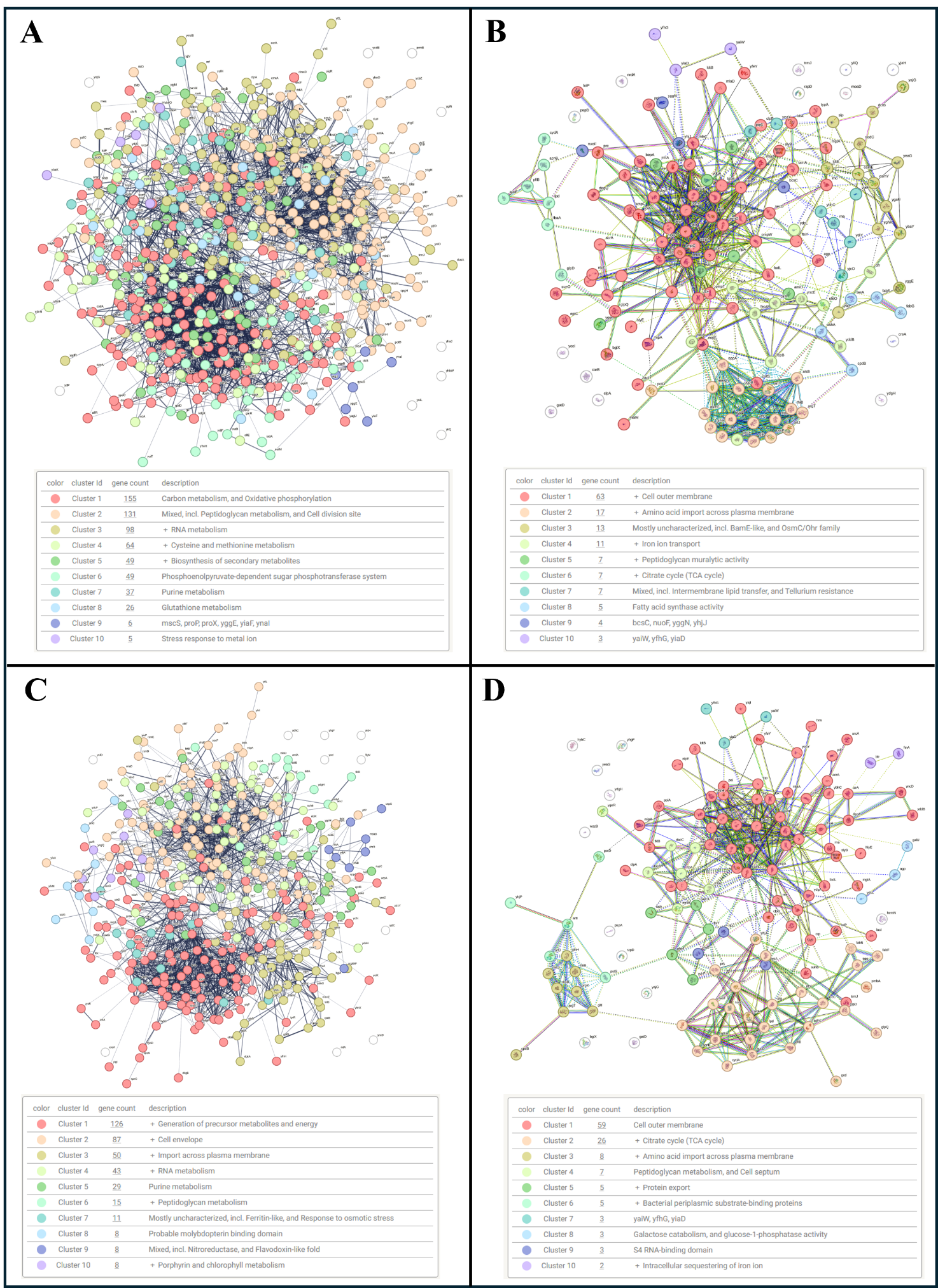


**Supplementary Figure 1: Functional protein association networks analysis performed by STRING.** Ten most representative functional clusters are shown for each sample. **A:** Cell lysates proteome of control *E. coli* BL21(DE3). **B:** OMVs proteome of control *E. coli* BL21(DE3). **C:** Cell lysates proteome of recombinant *E. coli* BL21(DE3). **D:** OMVs proteome of recombinant *E. coli* BL21(DE3).

**
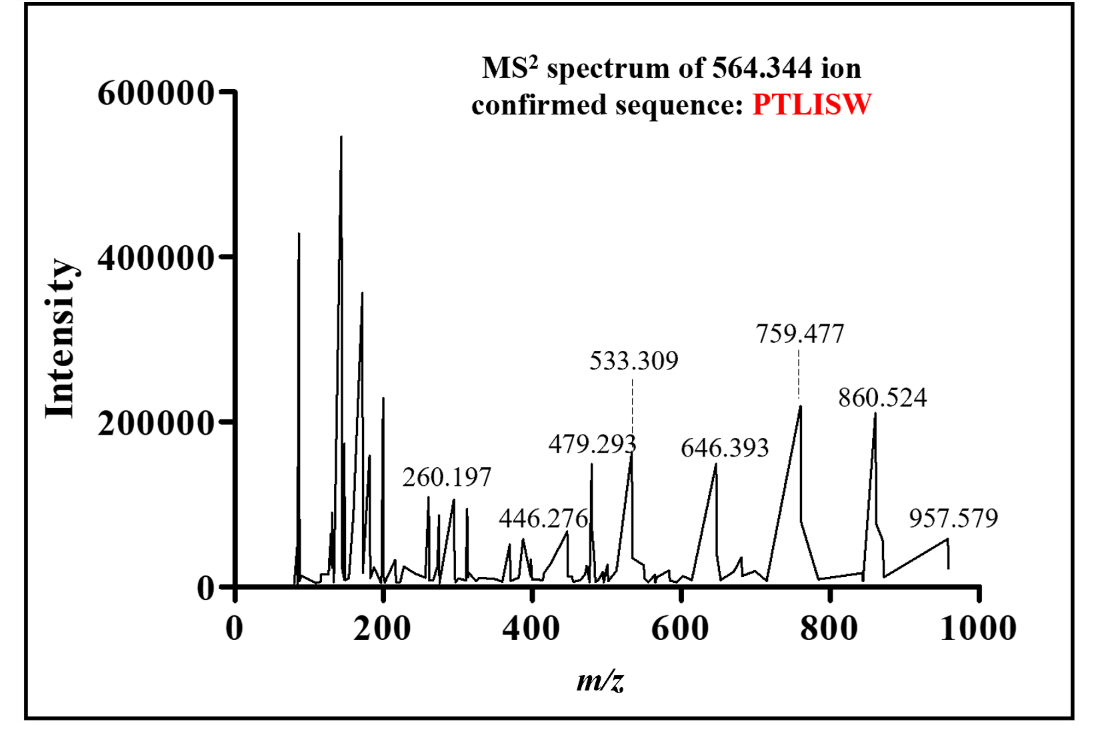
Supplementary Figure 2**

**Supplementary Figure 2 - MS^2^ spectrum of 564.344 ion found in ClyA-AR23 cell lysates.**

MS^2^ spectrum of the 564.344 ion found only in AR-23 samples, as shown in Figure 6A. MS signals confirmed the amminoacidic sequence reported in red, similarly to the MS^2^ spectrum of AR-23 purified peptide shown in Figure 5B. Raw data, obtained from Xcalibur QualBrowser, were reported in Graphpad Prism for visualization.


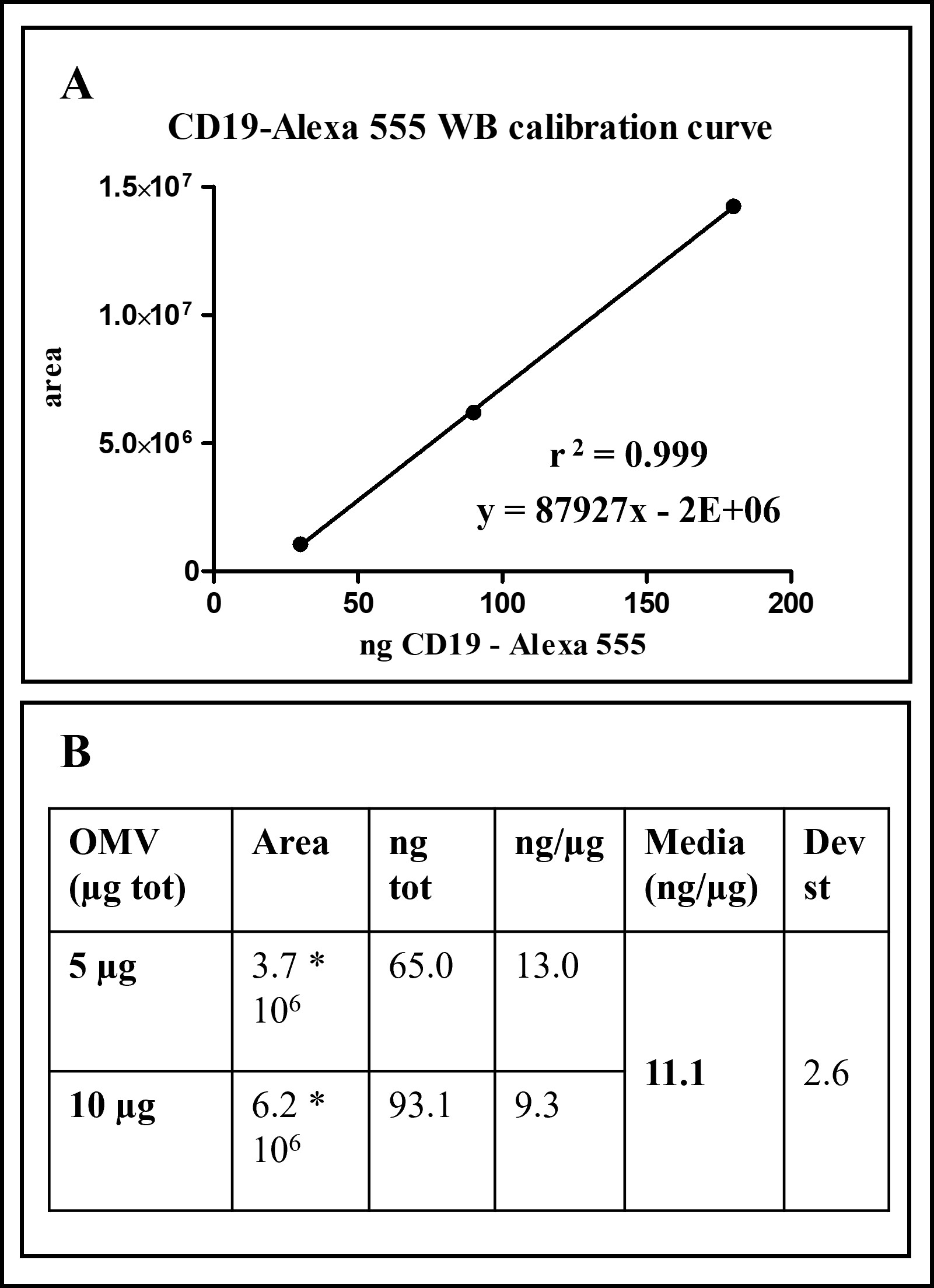


**Supplementary Figure 3: Densitometric analysis of ClyAhis in OMVs. A:** CD19-Alexa 555 calibration curve in SDS-PAGE (200ng, 100 ng and 25ng). **B:** ClyAhis protein amount compared to OMVs total proteins.
